# Molecular evolution of the *Pi-d2* gene conferring resistance to rice blast in *Oryza*

**DOI:** 10.3389/fgene.2022.991900

**Published:** 2022-09-06

**Authors:** Pengfei Xie, Jia Liu, Ruisen Lu, Yanmei Zhang, Xiaoqin Sun

**Affiliations:** Institute of Botany, Jiangsu Province and Chinese Academy of Sciences, Nanjing, China

**Keywords:** evolutionary history, origin, *Pi-d2*, rice blast resistance gene, selection

## Abstract

The exploitation of plant disease resistance (*R*) genes in breeding programs is an effective strategy for coping with pathogens. An understanding of *R* gene variation is the basis for this strategy. Rice blast disease, caused by the *Magnaporthe oryzae* fungus, is a destructive disease of rice. The rice blast resistance gene *Pi-d2* represents a new class of plant *R* gene because of its novel extracellular domain. We investigated the nucleotide polymorphism, phylogenetic topology and evolution patterns of the *Pi-d2* gene among 67 cultivated and wild rice relatives. The *Pi-d2* gene originated early in the basal Poales and has remained as a single gene without expansion. The striking finding is that susceptible *Pi-d2* alleles might be derived from a single nucleotide substitution of the resistant alleles after the split of *Oryza* subspecies. Functional pleiotropy and linkage effects are proposed for the evolution and retention of the disease-susceptible alleles in rice populations. One set of DNA primers was developed from the polymorphic position to detect the functional nucleotide polymorphism for disease resistance of the *Pi-d2* gene based on conventional Polymerase Chain Reaction. The nucleotide diversity level varied between different domains of the *Pi-d2* gene, which might be related to distinct functions of each domain in the disease defense response. Directional (or purifying) selection appears dominant in the molecular evolution of the *Pi-d2* gene and has shaped its conserved variation pattern.

## Introduction

Asian rice (*Oryza sativa* L.) is grown worldwide. The history of rice cultivation can be traced back to the Yangtze River basin in China 8,000–9,000 years ago (Doebley et al., 2009). Through long-term domestication and migration, two major types of *O. sativa—O. sativa* subsp. *indica* Kato and *O. sativa* subsp*. Japonica* Kato—have been derived from the wild rice species *Oryza rufipogon* Griff. and *Oryza nivara* Sharma et Shastry (Zhao et al., 2018). Rice is threatened by a variety of pathogens in its habitat (Santhanam et al., 2015; Teixeira et al., 2019). Rice blast, caused by the fungal pathogen *Magnaporthe grisea*, is one of the most devastating diseases of rice and results in annual losses of up to 157 million tons (Dean et al., 2012).

The plant innate immune system recognizes small molecules from pathogens using plant immune receptors. These receptors provide rapid pathogen defense and typically lead to hypersensitive response (HR) to limit pathogen spread (Kunze et al., 2004; Jones and Dangl, 2006). One family of plant immune receptors includes the receptor-like kinases (RLKs). These cell surface receptors are responsible for transducing ligand perception into intracellular signaling. RLKs consist of three functional domains, namely an ectodomain for ligand binding, a transmembrane domain and an intracellular kinase domain (Lu and Tsuda, 2021). The extracellular domains of RLK proteins are highly divergent and can bind different ligand molecules such as extracellular ATP, bacterial lipopolysaccharides, bacterial flagellin, elongation factor Tu (EF-Tu) or endogenous AtPep peptides (Couto and Zipfel, 2016). The kinase domain is relatively more conserved than the extracellular domain and plays a crucial role in transphosphorylation of both RLKs and their co-receptors once they form heterodimers in a ligand-dependent manner. The reciprocal transphosphorylation typically occurs at the activation loop, which is a regulatory region close to active sites (Chinchilla et al., 2006). Based on the conserved motif of the active site, RLKs can be further classified as RLKs with arginine-aspartate (RD) motifs that carry a conserved arginine residue just before an aspartate residue and non-RD kinases, which do not have conserved negatively charged arginine residue. The extracellular domain and intracellular domain characteristics are both considered in the classification of RLKs (Shiu and Bleecker, 2001).

The rice blast resistance gene *Pi-d2* is a single-copy gene located near the centromere of chromosome 6, and it encodes a protein belonging to the non-RD subclass of RLK. The *Pi-d2* gene that confers race-specific resistance to *M. oryzae* strain ZB15 was first cloned in the rice variety Digu (Chen et al., 2006). The putative protein of the *Pi-d2* gene presents an ectodomain carrying two motifs: a B-lectin domain relevant to ligand binding and a PAN domain associated with protein interactions. In addition, a single transmembrane domain and an intracellular serine-threonine kinase (STK) domain transduce ligand perception into intracellular signaling. A single amino acid at position 461 in the transmembrane region of Pi-d2 differentiates resistant alleles (461I) from susceptible alleles (461M). This is similar to Pi-ta, in which a single amino acid substitution*—*serine to alanine at position 918*—*is responsible for specific recognition of AVR-Pita (Bryan et al., 2000; Jia et al., 2000). Nucleotide polymorphisms responsible for the amino acid substitutions in Pi-d2 and Pi-ta are called functional nucleotide polymorphisms (FNPs). Each polymorphism differentiating the resistant and susceptible alleles can work as the basis of molecular markers. Sets of DNA primers were designed from polymorphism positions to detect FNP to facilitate crop breeding and improve disease resistance (Chauhan et al., 2002). Nevertheless, an effective marker for detection of the *Pi-d2* resistant alleles has not been developed.

Natural variations of the *Pi-d2* gene were analyzed in 41 rice accessions, including *O. sativa* and wild rice species (Li et al., 2015). A total of 13 nucleotide variations were identified in the open reading frames (ORFs) of *Pi-d2*, nine of which would result in nonsynonymous amino acid mutations. R7H, F116Y and H666R may be implicated in the conformation of functional domains of Pi-d2. In addition, nine haplotypes composed of seven resistant haplotypes and two susceptible haplotypes were identified. *Pi-d2* is an ancient gene that predates speciation of the rice subgroups (Li et al., 2015). However, the length of the open reading frame of *Pi-d2* was truncated at 2478 bp in this study, while Chen (2010) noted that an additional 20 amino acid residues should be embraced in the N-terminal of Pi-d2. Due to the limited number of wild rice species (*O. nivara* and *O. rufipogon*) studied by Li et al. (2015), the origin and evolutionary pattern of the *Pi-d2* gene remain ambiguous.

The U-box/ARM repeat protein OsPUB15 was reported as one of the PID2-binding proteins (Wang et al., 2015). Once the ectodomain of Pi-d2 binds a ligand, such as the elicitor from *M. oryzae*, the intracellular kinase domain forms homodimers and autophosphorylation. The ARM domain of OsPUB15 would be phosphorylated by Pi-d2 kinase to exhibit E3 ligase activity, which then acts with substrate downstream to finely regulate immune responses to maintain immune homeostasis (Wang et al., 2015). *FLAGELLIN SENSING 2* (*FLS2*) and *Xanthomonas resistance 21* (*XA21*), which are non-RD kinases, also interact with their respective E3 ligases to regulate plant defense responses (Song et al., 1995; Lu et al., 2011). Therefore, it has been suggested that the plant defense signaling pathway mediated by the interactions between non-RD kinases and E3 ubiquitin ligases broadly exists in plants, while conservation of the signaling pathway composed of interactions between a non-RD kinase and an E3 ligase in co-evolution has not yet been validated.

The large amount of pan-genome data available from diverse rice species or rice accessions offers us an opportunity to explore these questions via comparative analysis at the genomic level. In this study, we sampled 67 representative genome-scale data sets, covering all major species in *Oryza*, and identified homologous genes of *Pi-d2* and its interacting protein OsPUB15 in these genomes. We conducted a set of comparative analyses on their nucleotide polymorphism, phylogenetic topology and evolution patterns. These data clarify the origin and history of *Pi-d2* gene and its evolutionary relationship with *OsPUB15* in *Oryza*.

## Materials and methods

### Plant materials

A total of 67 accessions of cultivated and wild rice were selected for searching for homologues of the *Pi-d2* gene (Supplementary Table S1). The samples included 37 accessions of *O. sativa* ssp. *indica*, 16 of *O. sativa* ssp. *Japonica*, five of *O. rufipogon*, each one of *O. glaberrima* Steud., *O. punctata* Kotzchy ex Steud., *O. nivara*, *O. brachyantha* A. Chev. et Roehr., *O. glumipatula* Steud., *O. meridionalis* N.Q.Ng and *O. longistaminata* A. Chev.& Roehr. with *Leersia perrieri* (A.Camus) Launert as outgroup, all of which represented all of the major genetically distinct clusters in *Oryza* (Stein et al., 2018). Genome or pan-genome data were retrieved from the RicePanGenome database (http://www.ncgr.ac.cn/RicePanGenome) and the Rice Resource Center (https://www.ricerc.com) (Zhao et al., 2018; Qin et al., 2021).

### Identification of the *Pi-d2* Gene.

The amino acid sequences of Pi-d2 in Digu (GenBank: ACR15163.1) were used as a query to run a BLASTp search against all protein sequences in each genome. The threshold expectation value was set to 1.0. All hits were further subjected to Pfam analysis (E = 10^–4^) (http://pfam.sanger.ac.uk/) to confirm the presence of detectable domains of B-lectin, PAN and S-Tkc in each sequence. When two or more transcripts were annotated for a single gene, the longest form was selected. A similar strategy was used to identify OsPUB15 (LOC_Os08g01900) protein.

### Sequence alignment and phylogenetic analysis

Amino acid sequences of Pi-d2 were aligned using ClustalW with default options (Thompson et al., 1994) and then manually corrected in MEGA X (Kumar et al., 2018). Phylogenetic analyses were performed using IQ-TREE (version 2.2.0) with the maximum likelihood algorithm (Nguyen et al., 2015). ModelFinder was used to estimate the best-fit model of nucleotide substitution (Kalyaanamoorthy et al., 2017). Branch support values were calculated using SH-aLRT (Anisimova et al., 2011) and UFBoot2 (Minh et al., 2013) with 1000 bootstrap replicates. The phylogenetic tree of OsPUB15 was reconstructed using the same pipeline.

Based on the phylogenetic trees of Pi-d2 and OsPUB15, co-evolution analyses were conducted by MirrorTree (Ochoa et al., 2015). The server-generated scatter plots were derived from the branch lengths of a pair of corresponding species of two reconstructed phylogenetic trees.

### Sequence data analysis

DnaSP 5.10 software (Rozas et al., 2003) was used to calculate the values of *S* (number of polymorphic or segregating sites), *π* (nucleotide diversity), *θ* (theta from *S*, theta-W), nonsynonymous/synonymous substitutions (Ka/Ks) and *D* (Tajima’s D) (Tajima, 1989) and then to draw the sliding window of nucleotide diversity (*π*).

PopART1.7.1 was used to infer and visualize the genetic relationships among interspecific sequences of *Pi-d2* (Leigh and Bryant, 2015). All *Pi-d2* haplotypes from 10 species in *Oryza* were taken as input, and TCS methods were used to construct a haplotype network (Clement et al., 2000). The appearance of the network, including colors, fonts and positions of nodes and labels, was modified through a network viewer panel.

### Positive-selection analysis

Positive-selection analysis was performed on “The Selecton Server” (http://selecton.tau.ac.il/), and the *Ka*/*Ks* values for each of the amino acid residues of Pi-d2 were calculated. Five calculation models were used to identify the positive-selection sites under the query of *Pi-d2* DNA variants: M8 (positive selection enabled, beta + w ≥ 1) (Yang et al., 2000), M5 (positive selection enabled, gamma) (Yang et al., 2000), mechanistic-empirical combination (MEC model) (Stern et al., 2007), M8a (beta + w = 1, null model) (Swanson et al., 2003) and M7 (beta, null model) (Yang et al., 2000). The data were imported into Microsoft Excel 2007 for statistical analysis and sliding window drawing.

### Site-specific Polymerase Chain Reaction (PCR) of Pi-d2

Since the amino acid identified at position 461 of Pi-d2 differentiated the resistant Pi-d2 allele (461I) from the susceptible Pi-d2 alleles (461M) (Chen et al., 2006), a primer set of Pi-d2-S/Pi-d2-AS (5′-GCA​CAA​TAC​CAT​TAT​TAT​TGT​CAT​TAT​A-3’; 5′-GTT​GTC​GTC​AAG​TAG​AAC​ATT​CTC​A-3′) was designed to detect presence of the *Pi-d2* resistant alleles, with an expectation of positive results for the resistant alleles and no bands for the susceptible alleles.

Eighteen rice landraces widely cultivated in the Yangtze River area were chosen for validation of the site-specific PCR primer set of *Pi-d2* in populations (Supplementary Table S2). Total DNA of the samples was extracted from young leaflets by the CTAB method (Richards et al., 2001).

The site-specific PCR primer set of *Pi-d2* was used to verify eight samples of each of the 18 rice landraces. The reaction volume was 20 μL, and the components and final concentrations included 1.0 μL of DNA template (20 ng/μL), 10.0 μL of 2×reaction mix (containing 20 mM Tris-HCl, 100 mM KCl, 3 mM MgCl_2_, 400 μM dNTPs and bromophenol blue), 1.0 μL of each primer (10 mM) and 0.4 μL of Taq DNA polymerase (2.5 U/μL); double-distilled water was added to produce a final volume of 20 μL. The amplification program was performed as follows: 94°C for 5 min; 30 cycles at 94°C for 45 s, 58°C for 45 s, and 72°C for 1 min; and 72°C for 10 min. The PCR products were analyzed by electrophoresis on a 1% agarose gel that was run at 80 V for 30 min and stained with ethidium bromide. The products were visualized and photographed using a gel imaging system.

## Results

### Molecular variation at *Pi-d2*

The *Pi-d2* gene encodes a predicted protein of 845 amino acids with an estimated molecular mass of 90 kDa (Chen et al., 2006). The deduced amino acid sequence of Pi-d2 contains all the domain characteristics of an RLK including an extracellular domain (B-lectin), a transmembrane domain and an intracellular kinase domain (S-Tkc). We surveyed the DNA sequence variability of *Pi-d2* in 58 accessions including 37 of *O. sativa* ssp. *indica*, 16 of *O. sativa* ssp. *Japonica* and five of *O. rufipogon* (Supplementary Table S1).

The *Pi-d2* coding sequence reading frame was intact in all accessions. No indel polymorphisms were detected. The data revealed a total of 35 nucleotide polymorphisms while no nucleotide differences were fixed between *O. sativa* and *O. rufipogon*. Within the *Pi-d2* coding sequence, we detected 10 nonsynonymous (amino acid changing) polymorphisms (Table 1), including the 461th functional polymorphism previously reported (Chen et al., 2006).

**TABLE 1:** Polymorphic sites of *Pi-d2* in different rice accessions.

Domain	Position^a^	Reference in Nipponbare^b^	*O. sativa* ssp. *indica*	*O. sativa* ssp. *Japonica*	*O. rufipogon*	Frequency (%)^c^
—	45|15	C(Gln)	·/A(Lys)	—	—	1.7
—	126|42	A	·/T	·/T	—	—
—	150|50	T	—	—	·/C	—
—	290|97	C(Thr)	—	·/T(Met)	·	1.7
B-lectin	407|136	T(Phe)	—	—	·/A(Tyr)	1.7
—	426|142	C	—	—	·/T	—
—	437|146	G(Trp)	·/T(Leu)	—	—	5.2
—	513|171	G	·/A	—	—	—
—	539|180	A(Gln)	—	—	·/G(Arg)	1.7
—	552|184	T(His)	—	—	·/G(Gln)	5.2
—	555|185	G	·/A	—	·/A	—
—	933|311	G	—	—	·/A	—
—	1022|341	T(Leu)	C(Ser)	·/C(Ser)	C(Ser)	77.6
PAN	1059|353	T	—	—	·/C	—
—	1148|383	C(Gla)	—	—	·/T(Val)	5.2
—	1158|386	G	—	—	·/A	—
—	1167|389	G	·/A	·/A	—	—
TM	1383|461	G(Met)	A(Ile)	·/A(Ile)	A(Ile)	77.6
—	1557|519	G	—	—	·/A	—
S-TKc	1635|545	T	—	—	·/C	—
—	1767|589	G	—	—	·/A	—
—	1800|600	G	—	—	·/A	—
—	1821|607	T	—	—	·/C	—
—	5	—	—	—	—	—
1872|624	G	—	—	·/A	—	
—	1881|627	G	—	—	·/A	—
—	1992|664	T	—	—	·/C	—
—	2040|680	T	—	—	·/C	—
—	2058|686	C	·/T	·/T	·	—
—	2109|703	T	·	—	·/C	—
—	2184|728	C	·	—	·/T	—
—	2232|744	C	·/T	·/T	—	—
—	2409|803	G	—	—	·/A	—
—	2434|812	G	—	—	·/A	—
—	2436|812	A(Ala)	—	—	·/G(Gly)	5.2
—	2523|841	T	—	·/C	·	—

a *Position* Nucleotide | amino acid positions of the polymorphic sites in the coding region or protein of the *Pi-d2*, gene. Amino acid replacement mutations are indicated in brackets.

b *Reference in Nipponbare* The *Pi-d2*, sequence in Nipponbare serves as reference. The reference amino acids are in black brackets, and the substituted amino acids are in red brackets.

c *Frequency* Ratio of number of accessions with the nonsynonymous polymorphisms to the total number of accessions.

Among the nonsynonymous polymorphisms, four, one and one were located in the B-lectin domain, PAN-AP domain, and transmembrane region, respectively, while the remaining four polymorphisms were located in the unknown functional regions of *Pi-d2*. The amino acids change at position 341 exhibited a similar distribution pattern with the functional polymorphism (M461I) in all rice accessions surveyed, implying that substitution at the nucleotide position 1022 might also affect the function of *Pi-d2*.

**FIGURE 1:**
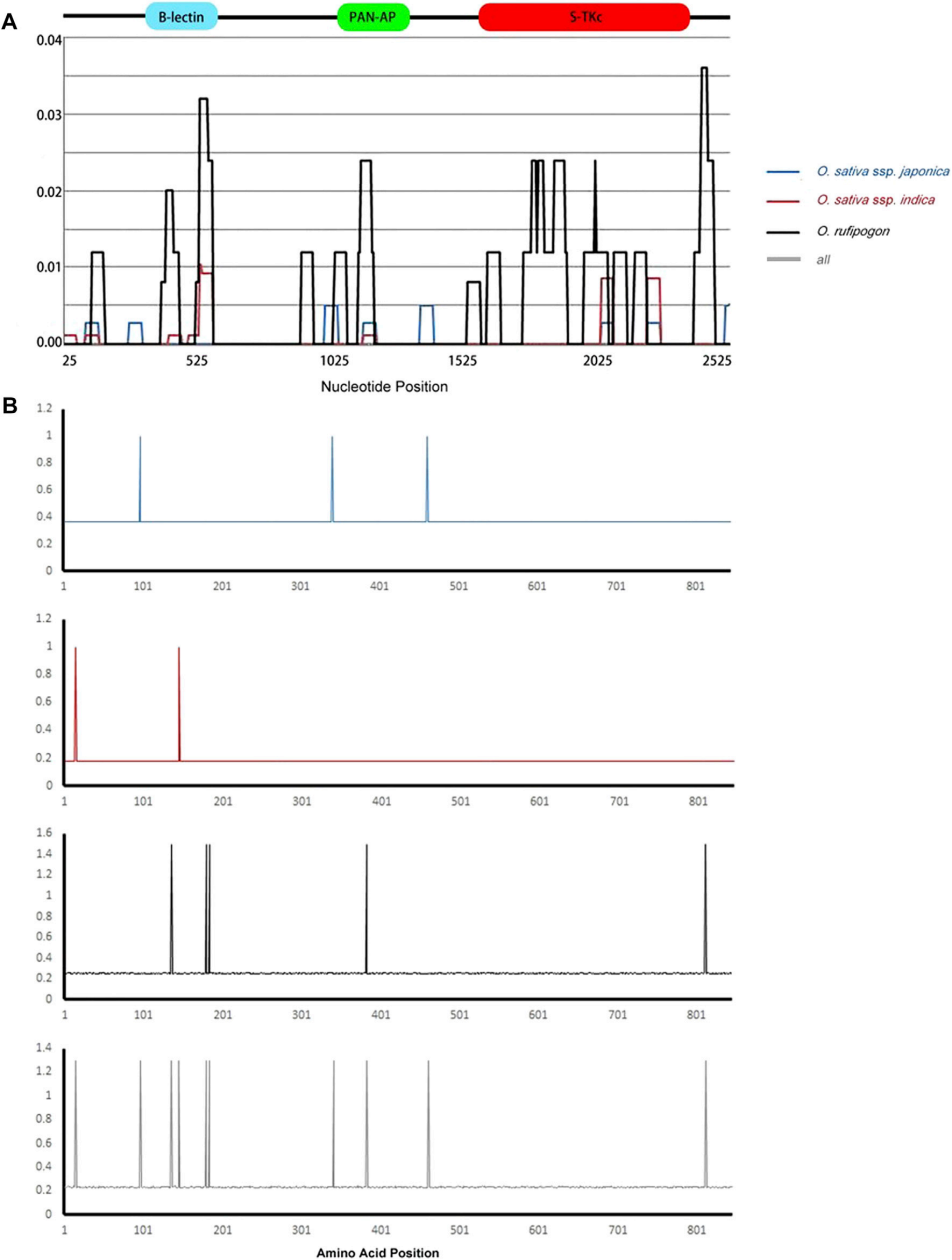
Sliding window of nucleotide diversity and positive-selection sites of *Pi-d2* in *Oryza sativa* ssp. *Japonica*, *O. sativa* ssp. *indica* and *O. rufipogon* subgroups. **(A)** The nucleotide diversity of *Pi-d2* in three subgroups. The *X*-axis represents the nucleotide position of *Pi-d2*; the *Y*-axis indicates value of nucleotide diversity per site. Values were assigned to the nucleotide at the midpoint of 5 bp. **(B)** The positive-selection sites of the Pi-d2 allelic variants under MEC model. The *X*-axis represents the position of the Pi-d2 amino acids; the *Y*-axis indicates the ratio of non-synonymous substitution (Ka) to the rate of synonymous substitution (Ks) (Ka/Ks); the B-lectin, PAN-AP, and S-TKc domains are marked on the corresponding region above the sliding windows. Structure of the *Pi-d2* coding region is shown at the top. *O. sativa* ssp. *Japonica*, *O. sativa* ssp. *indica*, *O. rufipogon* and the all group are represented by blue, red, black and gray lines, respectively.

The overall nucleotide diversity value (*π*) was 0.00182, and the *θ* value from segregating sites was 0.00298, indicating low diversity (Table 2) based on previously published criteria (Yang et al., 2008). A similar situation was also found in the *indica* subgroup (*π* = 0.00063, *θ* = 0.00076) and *japonica* subgroup (*π* = 0.00068, *θ* = 0.00107). The *O. rufipogon* (*π* = 0.00544, *θ* = 0.00454) showed a much higher level of diversity than these two subgroups. Therefore, the *Pi-d2* allelic variants of *O. sativa* presented a conservative evolutionary route during plant evolution, while *O. rufipogon* may have undergone an intermediate rate of evolution during host and pathogen co-evolution. This is reasonable as artificial selection would result in a 60–80% reduction in polymorphism in cultivated crops (Innan and Kim, 2004). The sliding window of *π* also indicated a higher nucleotide polymorphism in *O. rufipogon* than that in cultivated rice (Figure 1). Peaks of nucleotide diversity were mainly concentrated in three domains of Pi-d2, which is consistent with the distribution pattern of the polymorphic sites shown in Table 1.

**TABLE 2:** Nucleotide polymorphism of the *Pi-d2* allelic variants.

Group	Subgroup	Component	Location(nt)	*S* ^a^	*Π* ^b^	*θ* ^c^	Tajima’s *D* ^d^	*Ka*/*Ks*
All		Total	1–2,535	35	0.00182	0.00298	−1.28903	0.1046
		B-lectin	328–585	7	0.00327	0.00586	−1.13656	
		PAN-AP	1,057–1,314	4	0.00142	0.00355	−1.26412	
		S-TKc	1,579–2,382	12	0.00208	0.00324	−1.03055	
Cultivated rice	indica	Total	1–2,535	8	0.00063	0.00076	−0.48226	0.0201
		B-lectin	328–585	3	0.00220	0.00277	−0.45293	
		PAN-AP	1,057–1,314	1	0.00020	0.00092	−1.12863	
		S-TKc	1,579–2,382	2	0.00105	0.00059	−1.48379	
	japonica	Total	1–2,535	9	0.00068	0.00107	−1.33689	0.2406
		B-lectin	328–585	0	0	0	0	
		PAN-AP	1,057–1,314	1	0.00052	0.00199	−1.15945	
		S-TKc	1,579–2,382	2	0.00033	0.00077	−1.49051	
Wild rice	rufipogon	Total	1–2,535	24	0.00544	0.0454	1.47138	0.0705
		B-lectin	328–585	5	0.01008	0.00930	0.56199	
		PAN-AP	1,057–1,314	3	0.00698	0.00558	1.57274	
		S-TKc	1,579–2,382	10	0.00749	0.00599	1.78900	

a *S* number of polymorphic or segregating sites.

b *π* Nucleotide diversity.

c *θ* Watterson’s nucleotide diversity estimator based on silent site.

d Tajima’s *D* Tajima’s *D* statistic (Tajima 1989) based on the differences between the number of segregating sites and the average number of nucleotide differences.

Pi-d2 consists of three main domains—B-lectin, PAN-AP and S-Tkc (Chen et al., 2006)—and the nucleotide diversity of each of the domains was analyzed in this study. The B-lectin domain showed a higher level of nucleotide diversity than the PAN and S-Tkc domains except for in *O. sativa* ssp. *Japonica* (Table 2), suggesting the abundance of genetic variability in the B-lectin domain. Sliding window analysis showed a main peak in the B-lectin domain and a lower peak in the PAN-AP domain (Figure 1), indicating that the polymorphic regions were mainly concentrated in the B-lectin domain.

### Selection on *Pi-d2*

To examine the evolutionary dynamics of the *Pi-d2* alleles, natural selection was evaluated by Tajima’s *D* test. Tajima’s *D* value was −1.28903 (*p* > 0.05) with no statistical significance for the total (Table 2). Similarly, the statistical values were negative for both *indica* subgroup (Tajima’s *D* = −0.48226, *p* > 0.05) and *japonica* subgroup (Tajima’s *D* = −1.33689, *p* > 0.05). No significant departure from neutral expectations was observed for the *rufipogon* subgroup, though Tajima’s *D* value was 1.47138. Therefore, no selection effect was detected. While Tajima’s *D* in all rice accessions was not statistically significant, the level of synonymous divergence (*Ks*) exceeded that of nonsynonymous divergence (*Ka*) in all accessions of *O. sativa* and *O. rufipogon*. This indicated that the rice blast resistant gene *Pi-d2* may have undergone purifying selection (Table 2).

Positive-selection sites of the *Pi-d2* alleles were identified using the Selecton Server. The *Ka*/*Ks* values for each amino acid site in the deduced Pi-d2 proteins were calculated under five models (M8, M8a, M7, M5 and MEC). The Ka/Ks values of most sites (∼90%) were <1, suggesting that these sites were potentially subjected to purifying selection (Table 2; Figure 1). Few positive-selection sites (Ka/Ks > 1) with statistically significant results were found in the ORFs. The five amino acid sites under positive selection in *O. rufipogon* were F136Y, Q180R, H184Q, A383V and A812T. In the entire group, consisting of both cultivated and wild populations, the number of positive sites increased to 10, namely C15A, T97M, F136Y, W146A, Q180R, H184Q, T341C, A383V, M461I and A812T. These findings suggest that artificial selection during rice domestication might have played a role in evolution of the *Pi-d2* gene.

### Origin and evolution of the *Pi-d2* haplotypes

**FIGURE 2:**
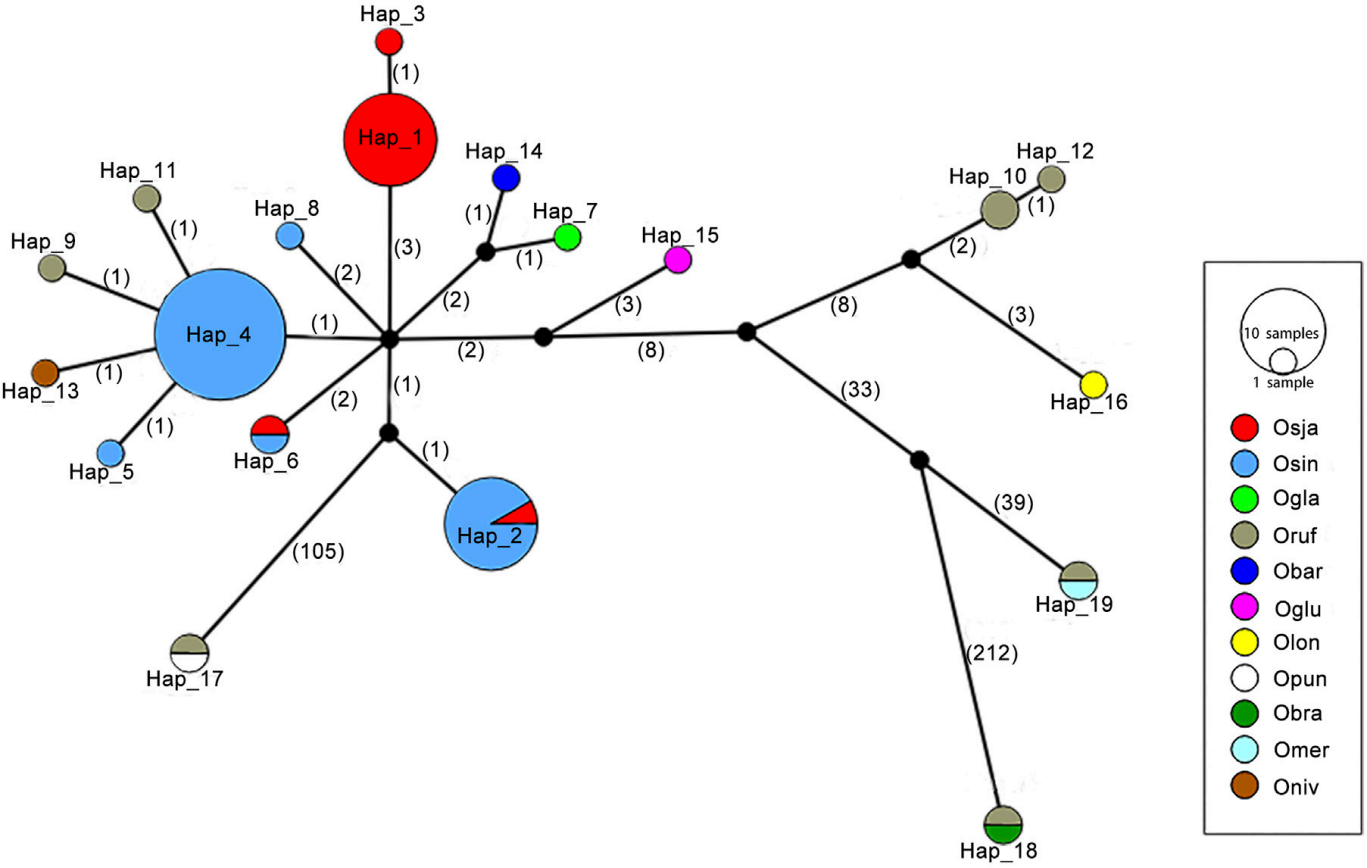
A haplotype network based on nucleotide polymorphisms of the *Pi-d2* coding region of 66 accessions of 11 *Oryza* species: *O. sativa* ssp. *indica*, *O. sativa* ssp. *Japonica*, *O. rufipogon*, *O. nivara*, *O. meridionallis*, *O. glaberrima*, *O. barthii*, and *O. glumaepatula*, *O. punctata* and *O. brachyantha*. Each group of haplotypes is shown as a solid circle. Each branch represents a single mutational step. Branches with small solid circles indicate that there is more than a single mutational step between haplotypes. A number next to a branch represents the length of the mutational steps. Different sizes of circles represent the different numbers of each haplotype.

A total of 19 *Pi-d2* haplotypes were identified from 11 *Oryza* species, including nine species with the AA genome, one species with the BB genome and one species with the FF genome (Figure 2; Supplementary Table S3). Among them, 17 haplotypes (89.47%) were identified exclusively in one *Oryza* species in the haplotype network, suggesting that the diversification of *Pi-d2* haplotypes occurred after the divergence of these *Oryza* species. The haplotype network also showed that the *Pi-d2* in cultivated rice might be derived from *O. rufipogon* and *O. nivara*. A total of seven haplotypes were identified in *O. sativa* ssp*. Japonica* and *O. sativa* ssp*. indica*, while only two haplotypes (Hap_2 and Hap_6) were shared between the two subspecies, suggesting that the haplotypes of *Pi-d2* evolved rapidly after the differentiation of the two subspecies.

All haplotypes identified in *O. rufipogon* and *O. sativa* ssp*. indica* were the resistant haplotypes (461I). All the haplotypes were exclusively identified in eight species of *Oryza*, namely *O. nivara*, *O. brachyantha*, *O. barthii*, *O. glumaepatula*, *O. meridionalis*, *O. longistaminata*, *O. punctata* and *O. glaberrima*, contained 461I as well. Only Hap_1 and Hap_3, the two prevalent haplotypes in *O. sativa* ssp*. Japonica*, were expected to be susceptible haplotypes (461M). These results suggest that the methionine-461 allele was recently derived from the ancestral *Pi-d2* alleles that carry isoleucine at 461.

### Phylogenetic analysis of *Pi-d2*

**FIGURE 3:**
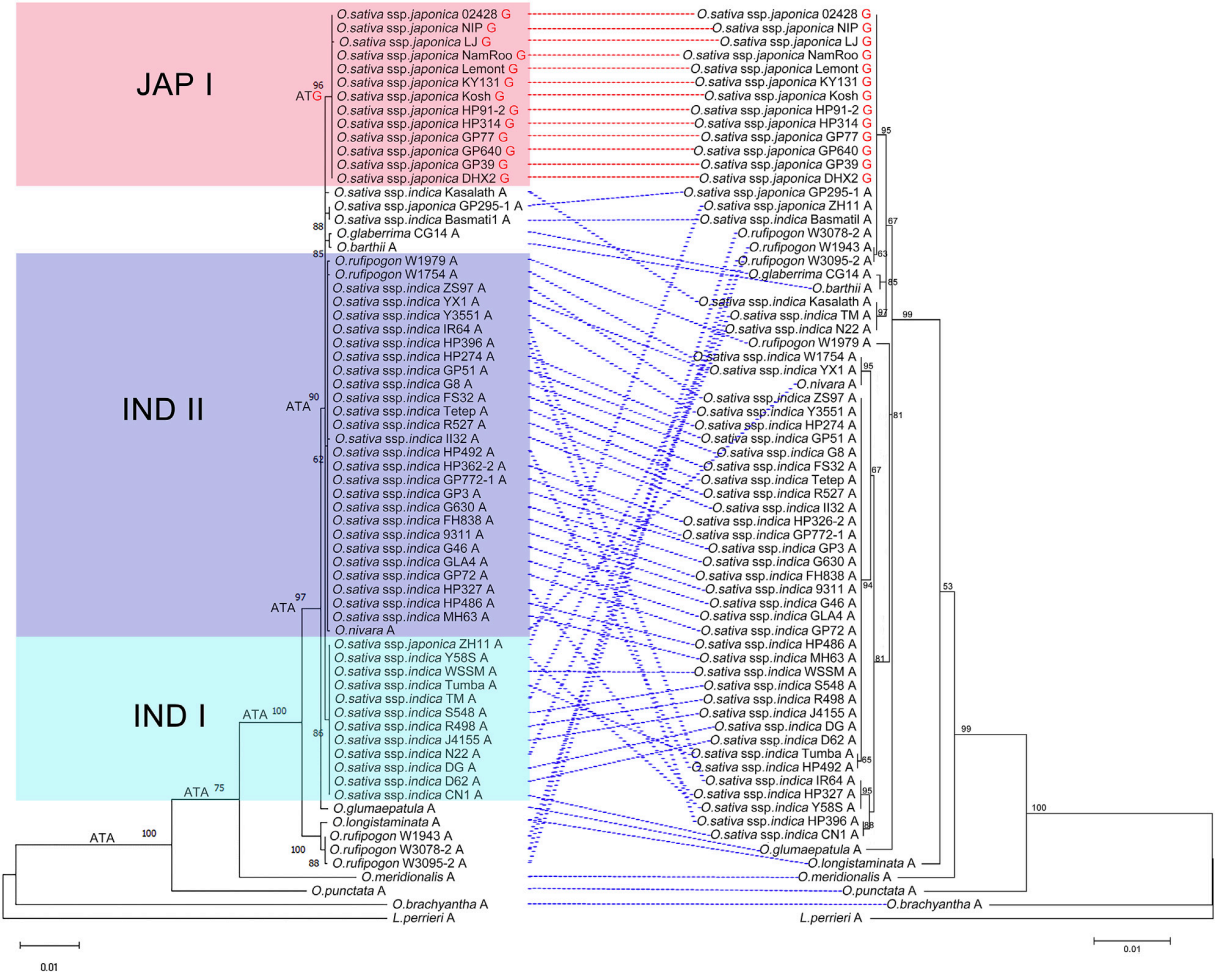
Reconstruction of the evolutionary history of *Pi-d2* (left) and *OSPUB15* (right) in *Oryza* with *Leersia perreri* as outgroup. The tree shows a set of inferred nucleotides (states) at the Pi-d2 polymorphic position 1383. Non-synonymous changes at the codon are depicted in black (ATA) or red (ATG) next to their corresponding node. Nucleotides (guanine in red and adenine in black) at position 1383 of *Pi-d2* in different accessions are indicated at the end of the name. *Pi-d2* and *OSPUB15* identified in the same accessions are connected by dotted lines (the assumed susceptible alleles are dotted in red, while the assumed resistant alleles are dotted in blue).

To better understand the evolutionary pattern of *Pi-d2* in *Oryza*, a phylogenetic tree was reconstructed based on 66 orthologs of Pi-d2 protein identified in 11 species of *Oryza* with *Leersia perreri* as the outgroup. Figure 3 shows that the topology of the *Pi-d2* tree was consistent with that of the *Oryza* species tree (Stein et al., 2018). The *Pi-d2* orthologs in *O. brachyantha* (FF), *O. punctata* (BB) and *O. meridionalis* (AA) were located in the outmost part of the phylogenetic tree. The *Pi-d2* orthologs in several accessions of *O. rufipogo*n and *O. longistaminata* grouped into one clade, sister to the other clade that included all the cultivated rice. The remaining accessions clustered in a monophyletic group were further divided into three major groups with strong support: one consisting of most accessions of *O. sativa* ssp. *Japonica* (Jap I), the second consisting of 11 accessions of *O. sativa* ssp. *indica* with the exception of ZH11 (*O. sativa* ssp. *japonica*) and the third group including 25 accessions of *O. sativa* ssp. *indica*, two accessions of *O. rufipogon* and *O. nivara*. These results revealed a close relationship of *Pi-d2* in *O. sativa* ssp. *indica* to that in both *O. rufipogon* and *O. nivara*.

It was reported that the single amino acid difference at position 461 of Pi-d2 distinguished resistant and susceptible alleles of *Pi-d2* (resistant: Ile; susceptible: Met; Chen et al., 2006). To learn more about the evolutionary trajectory of *Pi-d2*, we inferred the ancestral state of the nucleotide sequences coding for the polymorphic position. This analysis revealed that the Ile residue encoded by ATA is an ancestral state at this position and is still present in most *Pi-d2* sequences from wild *Oryza* species and *O. sativa* ssp. *indica* (Figure 3). A transition from ATA (coding for Ile) to ATG (coding for Met) occurred after the split of two *O. sativa* subspecies and has been maintained in *O. sativa* ssp. *Japonica*. This change provided the possibility of a nonsynonymous Ile to Met mutation by an ATA to ATG transversion, which occurred in the rise of the clade containing *O. sativa* ssp. *Japonica*. The Ile461Met polymorphism ultimately ended the resistance of *Pi-d2* to rice blast disease.

The E3 ligase OsPUB15 can interact directly with Pi-d2 and regulate plant cell death and blast disease resistance (Wang et al., 2015). The topology of the phylogenetic tree of OsPUB15 remained consistent with that of Pi-d2. Co-evolution analysis using MirrorTree showed that *Pi-d2* and *OsPUB15* experienced significant co-evolution (r = 0.837, *p* < 0.00005), and tight linkage of *Pi-d2* and *OsPUB15* was established early in the split of *Oryza* from *Leersia*. Thus, we assumed that *Pi-d2* and *OsPUB15* might be involved in plant disease resistance as a functional and co-evolved unit.

### Identification of the resistant alleles of *Pi-d2* by FNP-based molecular markers

Functional nucleotide polymorphism (FNP) at the nucleotide position 1383 of the *Pi-d2* gene distinguished the resistant and susceptible alleles and provided an opportunity to develop reliable molecular markers for identification of the resistant alleles of the *Pi-d2* gene to facilitate rice molecular breeding. Therefore, a primer set specific to the resistant *Pi-d2* alleles named Pi-d2-S/AS was developed to detect FNP for conventional PCR.

**FIGURE 4:**
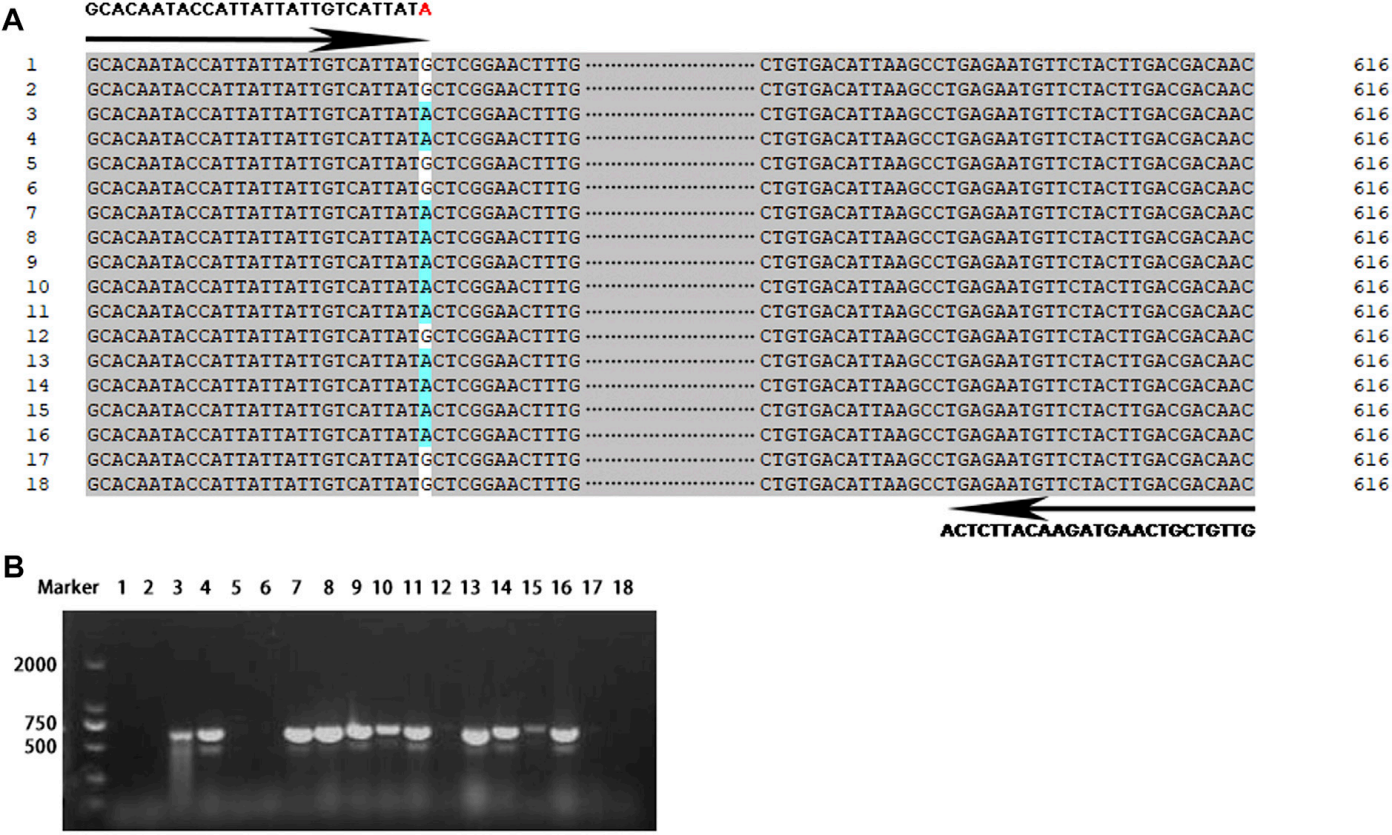
Development and validation of the FNP-based molecular marker. **(A)** Primer set (Pi-d2-S/AS) designed to target the FNP site that distinguished resistant and susceptible alleles with an expected PCR-amplified product of 616 bp. The numbers shown at the left of each sequence were referred to the landraces in Supplementary Table S2. The functional nucleotide polymorphic site of landrace 3, 4, 7–11, 13–16 was adenine **(A)** highlighted in blue. **(B)** PCR based validation of the primer set in eighteen landraces by PCR. M: marker. 1–18: landraces referred in Supplementary Table S2.

Eighteen rice landraces widely cultivated in the Yangtze River area were chosen for the development and validation of the site-specific PCR primer set of *Pi-d2*. Based on resequencing of the *Pi-d2* gene, we found that these 18 rice accessions showed nucleotide variations at position 1383 bp of the *Pi-d2* gene, seven of which exhibited A and the other 11 with G (Figure 4A). The site-specific PCR primer set was designed to target this site that differentiated the resistant alleles from the susceptible alleles. An expected fragment of 616 bp was amplified in seven landraces with A at position 1383 bp of the *Pi-d2* gene, whereas the specific band was not obtained in any of the other 11 landraces with G (Figure 4B). The results remained consistent across eight individuals of each landrace (Supplementary Figure S1), validating the accuracy and reliability of identification based on the FNP-based molecular marker.

## Discussion

### Origin and evolutionary pattern of the *Pi-d2* gene

The size of the RLK family is very small in the common ancestor of plant and animal lineages (Shiu and Bleecker, 2003), but the RLK family has expanded in the plant lineage. It is now reported to have approximately 440 members in the common ancestor of the flowering plants (Shiu et al., 2004). We searched for orthologs of *Pi-d2* against 135 genomes of the green lineage on Phytozome (Supplementary Figure S2; Wu et al., 2022; Saarela et al., 2018). Pi-d2 was identified in 18 of 24 species of Poales with the amino acid similarity higher than 80%. These data indicate that this gene originated early in the basal Poales and dated back to an early stage in Poales evolution. Notably, *Pi-d2* remained as a single gene, without expansion, in all clades surveyed.

Li et al. (2015) identified the *Pi-d2* allele in 50 rice accessions and concluded that nine *Pi-d2* haplotypes exist in rice germplasm. Here, a total of 19 *Pi-d2* haplotypes were identified from 67 cultivated and wild accessions. Thus far, rice germplasm without a *Pi-d2* homolog has not been identified, suggesting that the *Pi-d2* gene in *M. oryzae* plays an important physiological role in addition to resistance.

A single amino acid at position 461 of the Pi-d2 protein distinguishes resistant and susceptible alleles. The amino acid methionine (M) at 461 was found in the susceptible Pi-d2 allele, and isoleucine (I) at 461 was found in the resistant allele. In the present study, the isoleucine (I) at position 461 was found in all wild rice accessions and *O. sativa* ssp*. indica*, suggesting that the resistant *Pi-d2* allele is likely a derivative of alleles from wild rice relatives. Although no haplotypes were shared by the wild and cultivated accessions, the *O. sativa* ssp*. indica* was closely related to *O. nivara* and *O. rufipogon*, the ancestral progenitor of *O. sativa*, in the phylogenetic tree, indicating a complex evolutionary history of *Pi-d2* between *O. sativa* ssp*. indica* and its two wild relatives. Li et al. (2015) concluded that *Pi-d2* originated from *O. nivara*. However, our results revealed a more complex situation. Instead of a single origination of Pi-d2, uncoupling of differential domestication processes might have occurred over many rounds in these germplasm accessions. Two major haplotypes of *O. sativa* ssp*. indica* clustered separately in the phylogenetic tree, suggesting that the resistant alleles diverged recently after the formation of subspecies of *O. sativa* ssp*. indica*. The susceptible alleles with methionine (M) at 461 were only identified in *O. sativa* ssp*. Japonica* and thus were inferred to be derived from single nucleotide substitution of the resistant alleles.

Among all *R* genes, the most prevalent were the nucleotide-binding site plus leucine-rich repeat (NBS-LRR) genes. *Pi-d2* is a new class of rice *R* genes because of its novel extracellular domain. We analyzed the nucleotide polymorphisms of the *Pi-d2* gene in 67 geographically diverse rice accessions composed of 11 *Oryza* species to gain insight into the origin and evolution of *Pi-d2*. The low nucleotide diversity (0.182%) of the *Pi-d2* gene was comparable to the conserved category of the NBS-LRR gene (type I; *π* < 0.5%; Yang et al., 2008) and lower than the average diversity (0.32%, Yang et al., 2006) of housekeeping genes between Nipponbare and 93–11. In addition to the low nucleotide diversity, type I of the NBS-LRR gene contained only one copy in each of the cultivars just like the *Pi-d2* gene. This conserved evolutionary pattern may have been caused by artificial selection. Rice cultivars are the products of thousands of years of human selection. During crop improvement after domestication, humans exercised extremely strong selective pressure on target genes controlling key morphological and agronomic traits. Given that one gene was targeted by artificial selection, it would be feasible to expect there would be different levels of polymorphism between cultivars and wild rice. Indeed, polymorphism has been reduced by 60–80% (Innan and Kim, 2004) in cultivated crops. In this study, the nucleotide diversity was reduced by up to 80% in cultivated rice compared to the wild rice. Significant differentiation between cultivated and wild rice was also detected.

Unlike other target genes that are normally under directional selection, *R* genes may experience not only directional selection but also diversifying selection because of the temporal or geographic variation of pathogens (Stahl et al., 1999). Natural diversifying selection is characterized by the high levels of sequence polymorphism and an excess of nonsynonymous compared to synonymous substitutions (high *Ka*/*Ks*). It is impressive that the *Pi-d2* gene had an extremely low level of polymorphism and a value of *Ka* < *Ks*. These data suggest that directional (or purifying) selection is dominant in the *Pi-d2* gene. This is further supported by findings that *Pi-d2* may detect pathogen presence via an indirect mechanism (Chen et al., 2006).

### Evolution of the single amino acid polymorphism (I461M) in the *Pi-d2* gene

An amino acid substitution in a critical functional domain often results in a conformational change of the protein that may impact its biological function. A single amino acid substitution, serine (Ser) to alanine (Ala) at position 918, in the LRD of the Pi-ta protein determines the direct interaction with AVR-Pita and the resistance specificity to blast pathogen *M. oryzae* (Bryan et al., 2000; Jia et al., 2000). A single polymorphic residue, Glu230, is a major determinant of the increased cell death responses to the AVR-Pik effectors displayed by the *Pikm* NLR pair (De la Concepcion et al., 2021). For the *Pi-d2* gene, the ability to confer resistance to *M. oryzae* strain ZB15 can be attributed to a single amino acid change in the TM region.

To learn more about the evolutionary trajectory of *Pi-d2*, we inferred the ancestral state of the nucleotide sequences coding for the polymorphic position 1383. This analysis revealed that an Ile residue encoded by ATA is the ancestral state at this position and is still present in all *Pi-d2* sequences from wild *Oryza* species and most *O. sativa* ssp*. indica* cultivars. A transition from ATA (coding for Ile) to ATG (coding for Met) in position 1383 occurred after the split of *O. sativa* ssp*. indica* and *O. sativa* ssp*. Japonica*. This Ile461Met polymorphism ultimately abolished the function of Pi-d2. It is curious how a fairly minor difference can create such a major phenotype change.

The most striking finding was that the Ile (resistant) to Met (susceptible) change evolved recently in modern rice subspecies indicating that the susceptible *Pi-d2* alleles might have recently arisen. Remarkable differentiation between resistant and susceptible alleles was detected by our polymorphism data. This appears different from the *Pi-ta* and *Pi-k* genes in which the resistant alleles were derived from the susceptible ones.

It is unclear why rice populations evolve and retain disease-susceptible individuals. Functional pleiotropy is one plausible explanation. The *Pi-d2* gene, and especially the susceptible alleles, may have pleiotropic functions other than recognizing pathogens. In addition to the immune receptor function, some *R* genes are involved in signaling cascades important for additional cellular processes, such as drought tolerance, development and photomorphogenesis (Tameling et al., 2006). *Rps4* of *Arabidopsis* has not only conferred resistance to *Pseudomonas syringae* carrying the effector AvrRPS4 but also involved in *phyB* signaling (Gassmann et al., 1999). A relative lack of polymorphism was also found in *Rps4*, and only one amino acid polymorphism was detected in its LRR region (Bergelson et al., 2001; Bakker et al., 2006). Hence, we deduced that the *Pi-d2* susceptible alleles may be associated with other physiological functions in addition to resistance. Second, the emergence of the *Pi-d2* susceptible alleles in populations may be the consequence of genetic linkage. It is well established that physiological traits in plants are genetically linked (Ning et al., 2017). Therefore, if the *Pi-d2* susceptible allele is tightly linked to the other yield-increasing genes that are favored by the planters, the mutations of the Pi-d2 susceptible allele will therefore be fixed as a result of artificial selection on linkage effects within a short time. However, when traits are too tightly linked (for example within a genomic island), breeding strategies may be unable to uncouple them. In these cases, it is difficult to distinguish linkage effects from functional pleiotropy.

The patterns of the nucleotide variation are the basis of functional nucleotide polymorphism (FNP) for disease resistance. We designed one set of DNA primers from the polymorphic position to detect a single nucleotide polymorphism of the *Pi-d2* gene for conventional PCR and automated high-throughput DNA sequence analysis. This primer could be used to monitor the resistance of *Pi-d2* introgressions and facilitate efficient resistance breeding.

### Implication of evolutionary conservation in the function of the *Pi-d2* gene

*Pi-d2* encodes a predicted RLK protein, consisting of an N-terminal hydrophobic signal peptide, a B-lectin domain, a PAN domain, a TM domain and a C-terminal intracellular protein kinase domain. The extracellular lectin domain is proposed to bind possible pathogen-derived hydrophobic molecules such as plant products released by pathogen hydrolytic enzymes, or possible pathogen-specific molecules generated during infection (Barre et al., 2002). In this study, most of the nucleotide polymorphisms are located within the lectin domain, suggesting it is more variable than the other domains. Pi-d2 may detect pathogen presence via an indirect mechanism (Chen et al., 2006). However, the mode of action of this domain in the defense response remains unknown. The PAN motif appears to be involved in protein-protein and protein-carbohydrate interactions (Naithani et al., 2007). The transmembrane region is a critical region for preservation of LecRLKs’ function since a one aa change in this region leads to a non-functional Pi-d2 (Chen et al., 2006). The altered TM structure could not function properly in relaying ligand binding from the extracellular domain to the intercellular kinase catalytic domain. We found that almost all the *Pi-d2* alleles from *O. sativa* ssp. *Japonica* contain an I461M amino acid substitution, indicating the recent emergence of susceptible alleles after the split of two *O. sativa* subspecies. The kinase domain of RLKs is highly conserved in plants and animals (Shiu and Bleecker, 2001), and it is known to transduce downstream signals after forming a complex with the cell surface receptors (O’Neil and Greene, 1998). Pi-d2 contains a non-RD kinase domain, which is of particular interest since most plant RLKs, such as Xa21 (Song et al., 1995), Xa26 (Sun et al., 2004) and FLS2 (Lu et al., 2011), involved in disease resistance fall into this subcategory of kinases. The low nucleotide diversity and negative Tajima’s *D* of the S-Tkc domain in the *Pi-d2* gene suggest strong selective pressure, from pathogens or from biochemical constraints, to adopt and maintain non-RD kinases in activation of innate immune responses.

An important question in the process of plant resistance to pathogens is how to convert the recognition of pathogens into an effective immune response. Pattern recognition receptors (PRRs), mainly composed of RD kinase and non-RD kinase, play crucial roles. *Pi-d2* belongs to the non-RD subclass of RLKs, which is characterized by carrying an uncharged residue (such as cysteine, glycine, phenylalanine or leucine) in place of the conserved arginine (R) located just before the catalytic aspartate (D) residue (Dardick and Ronald, 2006).

Non-RD kinases have been associated with innate immune functions in both animals and plants (Dardick and Ronald, 2006). Compared to RD kinases, mechanisms for phosphorylation-mediated activation of plant non-RD complexes are largely unknown. A conformation change of non-RD kinase appears to be involved (Bender et al., 2021). A U-box/ARM repeat E3 ligase OsPUB15 was confirmed to interact directly with Pi-d2 and regulate plant cell death and blast disease resistance. In this study, co-evolution analyses revealed that *Pi-d2* and *OsPUB15* experienced significant co-evolution, and tight linkage of *Pi-d2* and *OsPUB15* was established in *Oryza* plants. Thus, we assumed that the linkage between *Pi-d2* and *OsPUB15* originated in early *Oryza* plant evolution. Besides *Pi-d2*, the well-characterized immune receptors, XA21 and FLS2, also belong to non-RD kinases (Dardick et al., 2012). Both XA21 and FLS2 interact directly with their respective plant E3 ligases to regulate plant defense responses (Wang et al., 2006; Lu et al., 2011). Thus, it was suggested that the signal pathway mediated by interactions between non-RD kinases and E3 ligases responsible for regulating innate immunity might broadly exist in the plant kingdom. Likewise, the *Arabidopsis* ETI receptors *RPM1*, *RPS2* and *RPS5* were found to all associate with *FLS2*, forming a larger signaling complex (Qi et al., 2011). Thus, non-RD kinase and ETI receptors may work in concert to trigger innate immunity.

The expansion of the RLK gene families in plants is highly uncoordinated. The genes involved in disease/stress resistance have undergone much larger expansion in the form of tandem duplications as opposed to the genes implicated in plant development (Vaid et al., 2012). *Pi-d2* was present as a single-copy gene in the genome under strong directional selection during evolution, unlike the RLK genes involved in disease/stress resistance but those in development. Overlapping functions of the same RLK gene in both development and stress tolerance are rare, suggesting differences in the activities of these genes (Vaid et al., 2012). It will be interesting to see if pleiotropy exists in the functions of the *Pi-d2* gene.

## Data Availability

The original contributions presented in the study are included in the article/Supplementary Material, further inquiries can be directed to the corresponding authors.
