# Supplementary figures and images for: Molecular evolution of the Pi-d2 gene conferring resistance to rice blast in Oryza
Source: Front Genet. 2022 Sep 6;13:991900. doi: 10.3389/fgene.2022.991900 (PMC9486079; doi:10.3389/fgene.2022.991900)

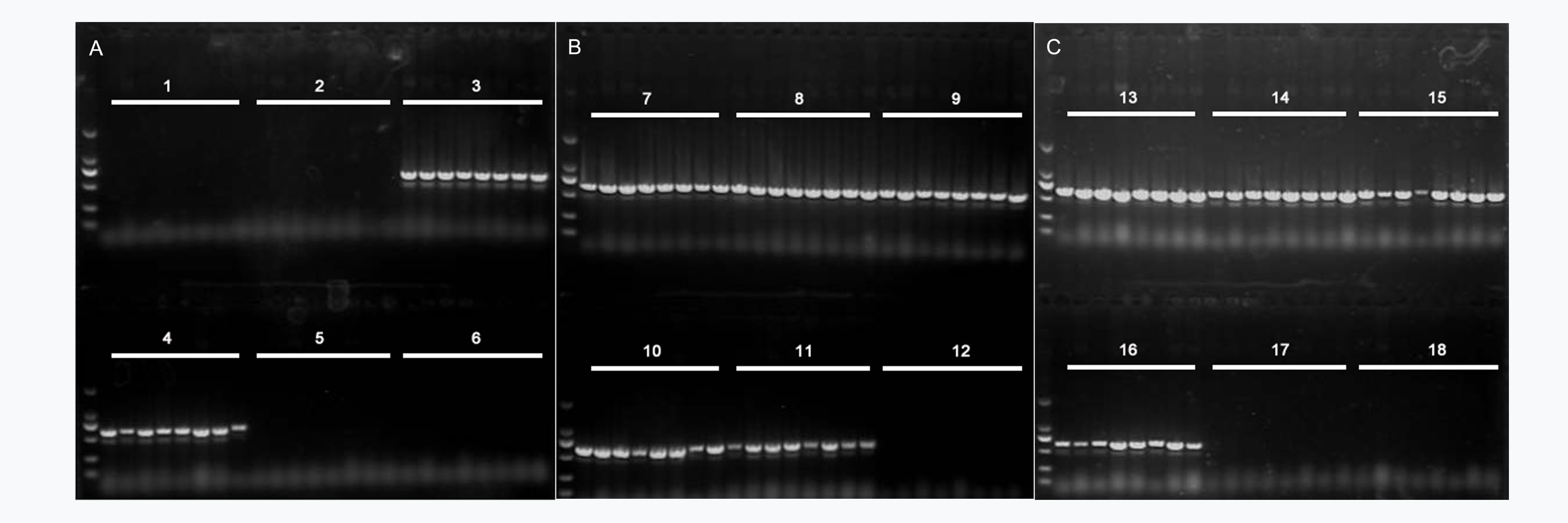

Supplement: Supplementary file 1 [file Image1.JPEG]

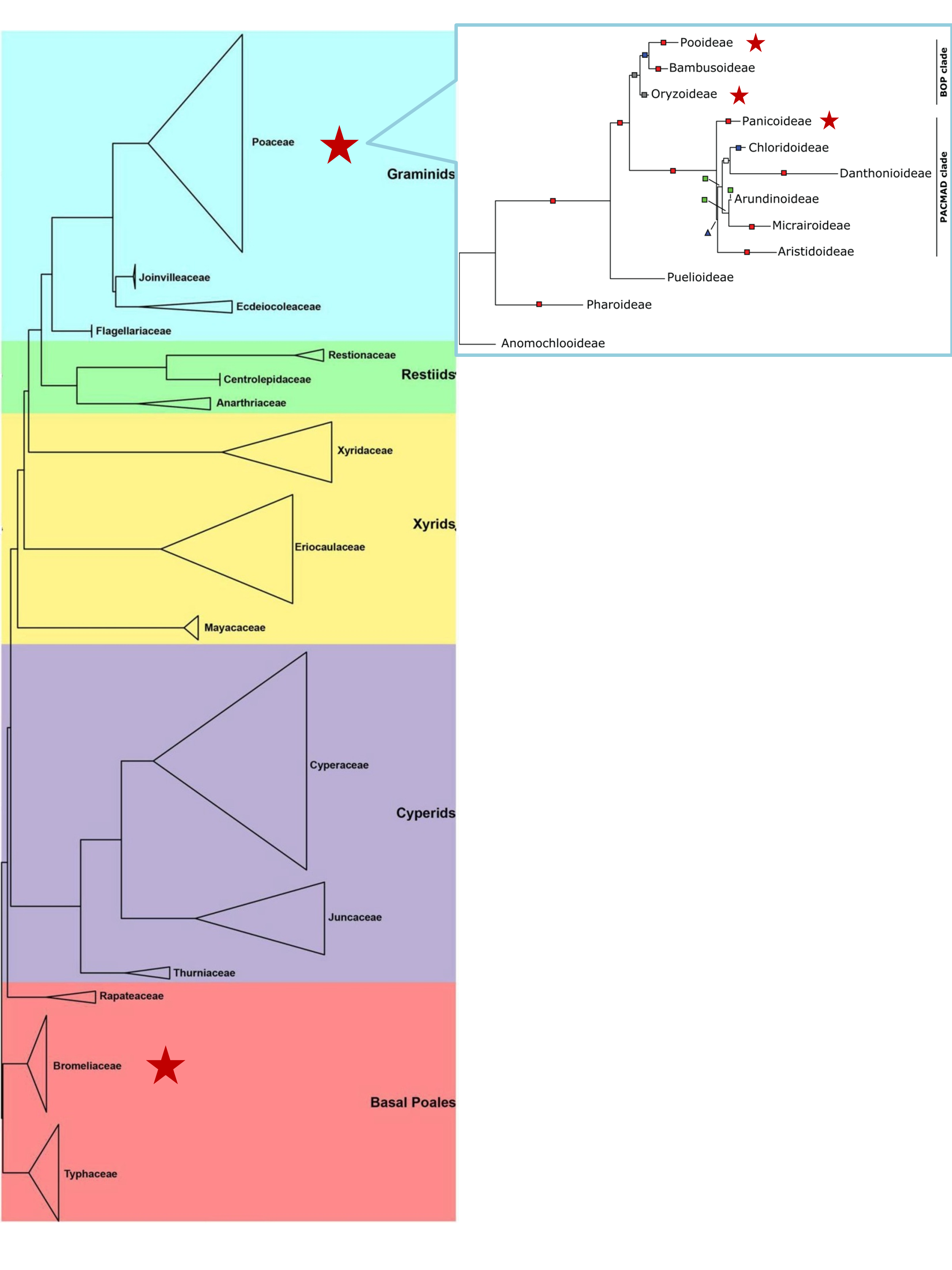

Supplement: Supplementary file 2 [file Image2.TIF]
